# Radiomics for the Diagnosis and Differentiation of Pancreatic Cystic Lesions

**DOI:** 10.3390/diagnostics10070505

**Published:** 2020-07-21

**Authors:** Jorge D. Machicado, Eugene J. Koay, Somashekar G. Krishna

**Affiliations:** 1 Division of Gastroenterology and Hepatology, Mayo Clinic Heath System, Eau Claire, WI 54703, USA; machicado.jorge@mayo.edu; 2 Department of Radiation Oncology, University of Texas MD Anderson Cancer Center, Houston, TX 77030, USA; ekoay@mdanderson.org; 3 Division of Gastroenterology, Hepatology and Nutrition, The Ohio State University Wexner Medical Center, Columbus, OH 43210, USA

**Keywords:** radiomics, quantitative imaging, texture, pancreatic cyst, intraductal papillary mucinous neoplasm

## Abstract

Radiomics, also known as quantitative imaging or texture analysis, involves extracting a large number of features traditionally unmeasured in conventional radiological cross-sectional images and converting them into mathematical models. This review describes this approach and its use in the evaluation of pancreatic cystic lesions (PCLs). This discipline has the potential of more accurately assessing, classifying, risk stratifying, and guiding the management of PCLs. Existing studies have provided important insight into the role of radiomics in managing PCLs. Although these studies are limited by the use of retrospective design, single center data, and small sample sizes, radiomic features in combination with clinical data appear to be superior to the current standard of care in differentiating cyst type and in identifying mucinous PCLs with high-grade dysplasia. Combining radiomic features with other novel endoscopic diagnostics, including cyst fluid molecular analysis and confocal endomicroscopy, can potentially optimize the predictive accuracy of these models. There is a need for multicenter prospective studies to elucidate the role of radiomics in the management of PCLs.

## 1. Introduction

The widespread use of high-resolution cross-sectional abdominal imaging has increased the incidental detection of pancreatic cystic lesions (PCLs) on imaging studies performed for unrelated reasons [1]. In a recent systematic review, the pooled global prevalence of asymptomatic PCLs was 8% (95% confidence interval, 4–14%), being higher in older subjects and when employing magnetic resonance imaging (MRI, 25%) compared to computed tomography (CT, 3%) [2]. Although the majority of PCLs are asymptomatic, mucinous PCLs (intraductal papillary mucinous neoplasms [IPMNs] and mucinous cystic neoplasms [MCNs]) are precursors of pancreatic ductal adenocarcinoma (PDAC). PDACs have a dismal survival rate, and are on the rise to become the second leading cause of cancer-related deaths in the United States [3]. Since up to 15% of PDACs arise from a mucinous cyst, their identification offers an opportunity for early detection of PDAC [4].

The diagnosis, risk-stratification, and management of PCLs have relied heavily on qualitative imaging characteristics and endoscopic ultrasound (EUS) with cyst fluid analysis (cytology, carcinoembryonic antigen [CEA], and amylase level) as a standard of care [5,6,7]. However, these diagnostic tools are suboptimal in the accurate differentiation of premalignant or malignant cysts, and hence there are multiple somewhat diverging expert guidelines from various international societies [5,7,8,9]. As evidenced in large surgical databases, 17–25% of patients who undergo surgical resection for a presumed mucinous lesion ultimately have a benign cyst [10,11]. The current standard of care is further inadequate at discriminating patients with mucinous PCLs that harbor advanced neoplasia (high-grade dysplasia [HGD] or PDAC) from those with low- or intermediate-grade dysplasia. Hence, the surgical decision is challenging and generally favors resection due to the mortality risk of PDACs, resulting in more than 60% of patients with mucinous PCLs who undergo resection and are found to have low or intermediate dysplasia on surgical pathology [11,12]. This overdiagnosis is problematic [13], because surgical resection for what are considered benign pathologies (e.g., low or intermediate grade dysplasia) can have dire consequences, with attendant risks of mortality (2%) and higher risks of morbidity (20–30%) that include exocrine and endocrine pancreatic insufficiencies [6].

In the last decade, significant progress in diagnostic tools has been made towards preoperative differentiation of PCLs and identifying mucinous cysts with advanced neoplasia. New endoscopic-based technologies, such as contrast-enhanced harmonic EUS (CH-EUS), through-the-needle microforceps biopsy, needle-based confocal laser endomicroscopy (nCLE), and cyst fluid molecular biomarkers, have shown promising results but require future validation in large prospective multicenter studies [14,15,16,17,18,19,20]. The field of radiology has also transformed to minimize the issues inherent to qualitative imaging interpretation, including high intra- and inter-observer variability, and limited utilization of all the imaging data. Cross-sectional CT scans and MRIs are integral in the management of PCLs. Radiomics, which involves extraction of high-dimensional data from cross-sectional imaging studies, is an evolving field in radiology that could potentially improve differentiation and risk stratification of PCLs. This review aims to depict this novel technology and its application in the management of PCLs, including assessing the current limitations and discussing future directions.

## 2. Definition of Radiomics

Radiomics, sometimes referred to as quantitative imaging, implies the extraction of a vast number of features from medical images, and its conversion to high-dimensional data [21]. Imaging features such as size, shape, physical transport properties, and texture are extracted from conventional CT, MRI, or positron emission tomography (PET) images into a database, and are then used to create statistical models that can improve diagnostic, prognostic, and predictive accuracy [22]. The principle of radiomics is that the gray-scale values of an image (i.e., pixels), and their spatial and temporal relationships, contain information on phenotype, pathophysiology, and biology (e.g., genomics, proteomics, and metabolomics) [23,24,25]. In addition, by using a quantitative, objective method and extracting large amounts of otherwise hidden imaging data, this technology offers an opportunity to circumvent some of the issues related to the traditional qualitative approach. Radiomics has been used to discern benign from malignant disease, and to prognosticate the clinical course of patients with lesions in a variety of organs (e.g., brain, pancreas, prostate, lung, liver, and kidneys) [22,26,27,28,29,30,31].

## 3. Process of Radiomics

**Figure 1:**
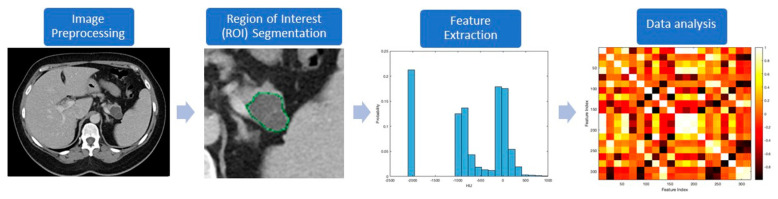
Diagnostic steps used in radiomics to evaluate a representative pancreatic cystic lesion (PCL).

The workflow processes in radiomics involve the following steps (Figure 1): (a) preprocessing, (b) segmentation, (c) feature extraction, and (d) data handling and analysis. Most of these processes are accomplished with computer software programs. Preprocessing follows imaging acquisition, and involves standardizing several parameters such as size of the pixel or voxels, number of the gray levels, range of gray level values, and signal intensity.

The process of segmentation consists of contouring the regions of interest (ROI), and represents a critical step because radiomic features will be extracted from these segmented volumes. Segmentation can be challenging due to the unclear margins of some lesions and lack of consensus about what to include—for example, the inclusion of normal pancreatic parenchyma in the analysis of PCLs. While this process can be conducted manually or automatically, both methods are subject to imprecision and reproducibility issues. Manual segmentation, usually conducted by experts, is tedious, time-consuming, and prone to inter-observer variability. In contrast, computer-aided methods may not reliably differentiate artifact and noise from the lesion itself. A combination of both approaches, in which automatic segmentation is performed first, followed by manual verification, might provide a more robust, reproducible, and consistent delineation of the ROI [32].

The next step, feature extraction, has the purpose of finding previously unseen patterns using computed automatic algorithms. Traditional nontexture features, which were created by human image processing experts, include size, shape, and location of a lesion. The texture analysis looks for the frequency distribution of pixels in a ROI (first-order features) and the relationship between pixels (second- or higher-order features), to evaluate properties such as smoothness, coarseness, and regularity (Table 1) [25,33].

Statistical analysis is performed after feature extraction. The initial step is changing the numeric values to a common scale and randomization of the data set. The number of variables (features) is then reduced to the lowest possible quantity to avoid over-fitting of the model by removing features with poor reproducibility and high correlation (“redundant” features) [21,34]. Statistical models can then be built using different algorithms (e.g., logistic regression, decision tree, k-nearest neighbors, neural networks, and deep learning) that include radiomic features with or without other predicting parameters. Model performance is measured using the area under the receiver-operating curve (AUC), accuracy, sensitivity, specificity, positive predictive value (PPV), and negative predictive value (NPV). Finally, these models can be internally validated during preliminary/single center studies but should ideally be externally validated in independent multicenter data sets [22].

The use of artificial intelligence (AI) offers a very powerful set of analytic tools to the massive amount of data extracted and used in radiomics [22]. Deep learning algorithms can be constructed to perform segmentation tasks, to extract hundreds of imaging features, and to directly analyze the imaging features using an automatic process without human intervention [35,36]. Consequently, the fields of radiomics and AI are being coupled to segment, extract, and analyze quantitative imaging data.

## 4. Literature Search

One of the authors (J.M.) conducted the electronic search. We searched MEDLINE via PubMed from inception through May 2020 to identify relevant studies. The search was limited to studies published using English language. The electronic search strategy was conducted on 25 June 2020 using a combination of phrases indicating the diseases of interest [“pancreatic cyst”, “pancreatic cystic lesion(s)”, “intraductal papillary mucinous neoplasm”, “mucinous cyst”, “serous cystadenoma”, “pancreatic cancer”] and imaging technology [“radiomic(s)”, “texture analysis”, “quantitative imaging”]. To identify additional potential studies, we reviewed the reference lists of the eligible primary studies and reviews.

## 5. Radiomics to Identify Cyst Type

A summary of the studies that have evaluated radiomic features in discriminating PCL type is presented in Table 2. Three recent studies have used radiomics with CT scans to differentiate serous cystadenomas (SCNs) from other types of PCLs. In a retrospective study of 260 patients who underwent pancreatic resection for a PCL between 2007–2017 in a single center from China, 409 radiomic features were evaluated to differentiate SCNs from other PCLs [37]. The use of 22 radiomic features (AUC 0.77) outperformed five clinical and standard imaging characteristics (AUC 0.71) to accurately diagnose SCNs preoperatively. In the independent validation cohort, the radiomic model yielded an AUC of 0.84, sensitivity of 67%, and specificity of 82%. In another study of patients with macrocystic SCNs (*n* = 26) and MCNs (*n* = 31) in surgical histopathology, 1,942 radiomic features were extracted [38]. A model with 18 high-order radiomic features (AUC 0.99) was far superior to using standard radiologic characteristics (AUC 0.77) in preoperatively differentiating macrocystic SCNs and MCNs. The third study including surgically resected patients with SCNs (*n* = 52) and MCNs (*n* = 25) also revealed that CT textural features are helpful in noninvasive differentiation of SCNs from MCNs (AUC from 0.66 to 0.77) [39].

The use of radiomics also appears to outperform conventional clinical and radiologic methods in discriminating between cyst types. In a study of 134 patients with a variety of histologically-confirmed PCLs, Dmitriev et al. found that manually selected quantitative features (location, intensity, and shape) in preoperative CTs more accurately classified cystic lesions than conventional clinical criteria (accuracy 80% vs. 62%) [40]. This study also found that the use of convolutional neural network features performed similarly to manual quantitative features, but the combination of both reached the highest accuracy (84%). Another study of 164 patients with postoperative pathological diagnosis of SCNs (*n* = 76), IPMNs (*n* = 48), and MCNs (*n* = 40) found that a model including 5 of 547 evaluated CT radiomic features with 4 clinical parameters achieved the highest accuracy to distinguish cyst types (84% in the training cohort and 80% in the validation cohort) [41]. None of the published studies evaluating the role of radiomics in discriminating cyst type has yet used MRI images. 

## 6. Radiomics to Advanced Neoplasia in IPMNs

Only a few studies have evaluated the role of radiomics in differentiating IPMNs with advanced neoplasia from indolent lesions with low-grade or intermediate dysplasia (Table 3). All these studies were retrospective; they had small sample sizes, were conducted at single centers, and only included patients with IPMNs confirmed in surgical pathology and with available preoperative images.

### 6.1. Role of CT

Most of the studies evaluating radiomics in IPMNs have used CT scans. The first study by Hanania et al. evaluated the performance of 360 imaging features using pancreas protocol CTs (arterial phase) of 53 patients with IPMNs who underwent surgical resection between 2003 and 2011 at MD Anderson Cancer Center (Texas, US) [42]. A total of 14 second-order texture features accurately distinguished IPMNs with low- from high-grade dysplasia on univariate analysis, with individual AUCs ranging from 0.64 to 0.82. The final panel consisted of 10 highest-performing features, which resulted in an AUC of 0.96, sensitivity of 97%, and specificity of 88%. Using this radiomic panel would have resulted in fewer patients with low-grade IPMNs who underwent an unnecessary surgery (12%), compared to the 2012 Fukuoka guidelines (36%).

In another study by Permuth et al. of 38 patients with IPMNs resected between 2006 and 2011 at the Moffit Cancer Center (Florida, US), a total of 112 features were extracted from portal-venous phase CT images [43]. Univariate analysis revealed 14 features that detected IPMNs with high-grade dysplasia with an AUC that ranged from 0.69 to 0.79. The diagnostic performance of the 14 features when combined (AUC 0.77) was better than using worrisome features as defined by the 2012 Fukuoka guidelines (AUC 0.54) and clinical parameters alone (AUC 0.73), but was lower than using Fukuoka guidelines specified high-risk PCL features (AUC 0.84) and blood expression of 5 mi-RNAs (AUC 0.83). Integration of radiomic features with mi-RNA improved the model performance to AUC of 0.92, and the addition of worrisome PCL features further improved the AUC to 0.93.

More recently, a study of 103 patients with branch-duct IPMNs operated between 2005 and 2015 at Memorial Sloan Kettering Center (New York, US) assessed 255 radiomic features using portal-venous phase CTs [44]. Prediction of high-grade dysplasia in IPMNs was suboptimal with clinical and standard radiologic parameters (AUC 0.67); however, application of radiomic features, either alone (AUC 0.76) or in combination with clinical data (AUC 0.79), increased the diagnostic accuracy. In a separate study, the investigators used the same patient cohort to evaluate the predictive performance of 4 novel radiographic features (enhanced boundary fraction, enhanced inside fraction, filled largest connected component fraction, and average-weighted eccentricity) and compared them with texture features [45]. The model performed best when combining the novel features with clinical covariates (AUC 0.81) or with texture features (AUC 0.78), rather than using the novel features (AUC 0.77) or texture features alone (AUC 0.74). The same research group recently compared the combination of 13 texture features, with cyst fluid protein markers predictive of high-grade dysplasia [46] in 33 patients with IPMN [47]. Radiomic features (AUC 0.83) outperformed proteomic analysis (AUC 0.74); however, the combination of both methods predicted high-grade lesions better (AUC 0.88). 

### 6.2. Role of MRI

There is paucity of information regarding the use of radiomics with MRIs in PCLs including IPMNs. Some investigators have attempted to discriminate high-grade from low-grade dysplasia in IPMNs using apparent diffusion coefficients (ADC) calculated with diffusion-weight images (DWI). These studies used a modified process of radiomics, in which only ADC summary metrics are extracted, rather than including the full array of potential radiomic features. Most of these studies used a single-slice ROI and found that lower ADC values were associated with high-grade dysplasia in IPMNs [48,49,50,51]. Hoffman et al. evaluated the utility of whole lesion ADC metrics among 18 patients with pathologically proven branch-duct IPMNs diagnosed between 2006 and 2015 in New York University [52]. Quantitative ADC metrics such as entropy (AUC 0.86), mean of the bottom 10th percentile (AUC 0.81), and mean of the 10–25th percentile (AUC 0.79) outperformed other ADC characteristics and standard radiologic findings. In a multivariate analysis with all ADC and standard features, entropy was the only significant predictor of high-grade dysplasia in IPMN.

## 7. Limitations of Radiomics

Radiomics is still an early discipline. While radiomic analysis is a tool that uses big data to identify correlations, it does not to provide a definitive diagnosis or establish malignant potential through imaging alone. Some of the challenges that have hindered translation of this approach into clinical practice include technical complexity; clinical research limited to expert institutions; and lack of standardization for segmentation, feature extraction, and reported metrics [21,23]. One of the biggest problems precluding multicenter collaborations has been that the acquisition techniques and processing methods used to generate the images vary significantly across institutions. Changes in parameters such as current, voltage, slice thickness, and reconstruction algorithms can impact image noise, pixel intensity, and relationship between pixels, and therefore might impair the reliability and reproducibility of radiomic models [53,54,55]. Although challenging, one possible solution to this problem is using uniform imaging acquisition protocols across institutions. An alternative would be applying harmonization corrections and generating stable models that account for imaging protocol peculiarities [56]. Overcoming these limitations will be of paramount importance to allow data sharing across multiple sites and to conduct large external validation studies.

Specifically in the field of PCLs and IPMNs, previous studies used heterogeneous imaging modalities, different inclusion/exclusion criteria, and variable radiomic features, which makes it difficult to combine and compare results in a meta-analysis. The retrospective and single-center nature of these studies also increases the risk of biased results and internal validity. Most studies had small sample sizes, raising concern for low power and over-fitted predictive models. This is despite the fact that most studies were conducted in large referral centers and participants were enrolled over long periods (~10 years). An explanation for this is that that reference histologic standard is surgical pathology, and only a limited number of patients with PCLs need resection. Adding autopsy data or using other endpoints for nonsurgical patients might enrich the sample sizes and increase the power of future studies. Another limitation of prior research is the lack of validation of the proposed models in independent datasets, so external validity and generalizability of their results are not ensured. In addition, to the best of our knowledge, the field of radiomics has not been applied to ultrasonographic or endonosonographic images of pancreatic cysts. Finally, the vast majority of previous studies used CT imaging, which cannot be extrapolated to other imaging modalities such as MRI.

## 8. Conclusions and Future Directions

The field of radiomics has the significant potential to inform the appropriate management of PCLs using an objective, noninvasive, and low-cost platform. Early studies have consistently shown better performance of CT radiomic features in differentiating PCLs compared to clinical and qualitative imaging interpretation, but more validation will be needed to ensure broad applicability and reproducibility. Additionally, future studies need to directly compare radiomic features with more invasive novel techniques such as fluid molecular biomarkers and EUS-nCLE, which have been shown to improve the distinction between mucinous and nonmucinous PCLs. However, EUS is not always indicated or performed in all PCLs, so even if radiomic features are not superior to other endoscopic-based technologies, they may still be valuable to noninvasively differentiate PCLs. 

More importantly, and despite the limitations of previous studies, radiomic models appear to outperform international consensus guidelines in accurately identifying IPMNs with advanced neoplasia. Furthermore, the integration of radiomic features with clinical variables and other diagnostic parameters (blood mi-RNA, cyst fluid protein markers) has demonstrated improved predictive accuracy of the models compared to using radiomics alone. While future multicenter prospective studies are needed to validate these findings with CT and MRI images, the impact of combining radiomic features with other novel methods such as cyst fluid molecular markers, EUS-nCLE, and CH-EUS should be tested as a possible multimodal approach that might increase the diagnostic accuracy of individual methods and might lead to the reduction of unnecessary pancreatic surgeries for PCLs. For this purpose, a multidisciplinary collaboration involving experts in radiology, gastroenterology, pathology, bioinformatics, and statistics is needed. Finally, local institutions need to envision dedicated teams with expertise in AI that might allow a transition from a qualitative radiologic assessment of PCLs into a new paradigm of quantitative imaging in clinical practice. 

## Figures and Tables

**Table 1:** Summary of most frequently used radiomic features.

Category	Features
Traditional nontexture features	Size (area, volume, major, and minor axis length, surface area) Shape (elongation, flatness, sphericity, and spherical disproportion) Location of a lesion
First-order texture features	Mean gray-level intensity Energy Entropy Standard deviation Skewness Kurtosis
Higher-order texture features	Gray-level co-occurrence matrix Gray-level run length matrix Gray-level size zone matrix Autoregressive model

**Table 2:** Summary of studies evaluating the diagnostic role of radiomics on discrimination of PCL type.

Author, Location, Year	Primary Outcome	Inclusion Criteria	Number of Patients	Image Type	Number of Radiomic Features	Best Model	Performance Training Set	Performance Internal Validation Set
Wei, China, 2019	Distinguish SCN vs. other PCLs	Surgically resected PCL (2007–2016)	260 (102 SCN, 74 IPMN, 35 MCN, 49 SPN)	CECT	409	22 radiomic features	AUC: 0.77 Sn: 69% Sp: 71%	AUC: 0.84 Sn: 95% Sp: 81%
Xie, China, 2020	Distinguish SCN vs. MCN	Surgically resected SCN or MCN (2010–2019)	57 (26 SCN, 31 MCN)	CECT	1,942	18 high-order radiomic features	AUC: 0.99 Sn: 94% Sp: 96%	--
Yang, China, 2019	Distinguish SCN vs. MCN	Surgically resected SCN or MCN (2013–2018)	77 (52 SCN, 25 MCN)	CECT	--	5 radiomic features	AUC: 0.77 Sn: 95% Sp: 83%	AUC: 0.66 Sn: 86% Sp: 71%
Dmitriev, USA, 2017	Discriminate PCL type	Surgically resected PCL	134 (74 IPMN, 14 MCN, 29 SCN, 17 SPN)	CECT	--	Random forest and CNN	Accuracy: 84%	--
Shen, China, 2020	Discriminate PCL type	Surgically resected PCL (2014–2019)	164 (76 SCN, 48 IPMN, 40 MCN)	CECT	547	5 radiomic + 4 clinical features, using random forest	Accuracy: 84% Precision IPMN: 86% Precision MCN: 82% Precision SCN: 85%	Accuracy: 80% Precision IPMN: 90% Precision MCN: 90% Precision SCN: 72%

PCL: pancreatic cystic lesion; SCN: serous cystadenoma; IPMN: intraductal papillary mucinous neoplasm; MCN: mucinous cystic neoplasm; SPN: solid pseudopapillary tumor; CECT: contrast-enhanced computed tomography; AUC: area under the curse; Sn: sensitivity; Sp: specificity; CNN: convolutional neural network.

**Table 3:** Summary of studies evaluating the role of radiomics on differentiating IPMNs with and without advanced neoplasia.

Author, Location, Year	Inclusion Criteria	Number of Patients	Image Type	Number of Radiomic Features	Best Model	Performance Training Set	Performance Internal Validation Set
Hanania, USA, 2016	Surgically resected IPMN (2003–2011)	53 (34 HGD, 19 LGD)	CECT	360	10 radiomic features	AUC: 0.82 Sn: 85% Sp: 68% (*)	AUC: 0.96 Sn: 97% Sp: 81%
Permuth, USA, 2016	Surgically resected IPMN (2006–2011)	38 (20 HGD, 18 LGD)	CECT	112	14 radiomic features +blood 5 mi-RNAs	AUC: 0.92 Sn: 83% Sp: 89% PPV: 88% NPV: 85%	AUC: 0.87
Attiyeh, USA, 2019	Surgically resected BD-IPMN (2005–2015)	103 (27 HGD, 76 LGD)	CECT	255	Radiomic + clinical features	AUC: 0.79 Sn: 71% Sp: 82% PPV: 95% NPV: 79%	--
Harrington, USA, 2020	Surgically resected IPMN	33 (7 HGD, 26 LGD)	CECT	13	Radiomic features + cyst fluid protein markers	AUC: 0.88 Sn: 71% Sp: 92% PPV: 71% NPV: 92%	--
Hoffman, USA, 2017	Pathology proven BD-IPMN (2006–2015)	18 (8 HGD, 10 LGD)	MRI with DWI	--	Entropy	AUC: 0.86 Sn: 100% Sp: 70%	--

* Estimates obtained with the highest performing individual radiomic feature. IPMN: intraductal papillary mucinous neoplasm; BD: branch-duct; HGD: high-grade dysplasia; LGD: low-grade dysplasia; CECT: contrast-enhanced computed tomography; DWI: diffusion weighted imaging; AUC: area under the curse; Sn: sensitivity; Sp: specificity; PPV: positive predictive value; NPV: negative predictive value.
